# Supplementary material for: Consent to minimally invasive tissue sampling procedures in children in Mozambique: A mixed-methods study
Source: PLoS One. 2021 Nov 8;16(11):e0259621. doi: 10.1371/journal.pone.0259621 (PMC8575303; doi:10.1371/journal.pone.0259621)
Supplement: S1 Appendix — (PDF) [file pone.0259621.s001.pdf]

cism centro de investigação em saúde de manhiça	*Centro de Investigação em Saúde da Manhiça (CISM)*<br>**ESTUDO DE VIABILIDADE E ACEITABILIDADE CADMIA**<br>**GUIÃO DE ENTREVISTA PARA FAMILIARES DE UM FALECIDO A 0-24 HORAS APÓS DA MORTE**	CaDMIA

Instruções

1. Apresente-se e dê as condolências.
2. Explique que:
   - Esta entrevista tem como objectivo explorar a sua experiencia recente com a morte e a sua opinião sobre a realização de MIAs (biopsias/colheita de amostras).
   - Foi convidado/a para esta entrevista porque a sua contribuição é essencial para entender o fenómeno da morte desde a perspetiva da família.
   - Vamos falar de questões relacionadas com a causa de morte e a possibilidade de realizar MIAs a entes-queridos. Espera-se que a entrevista dure 10-15 minutos.
3. Diga que, se permitir, a entrevista será gravada, somente para não corrermos o risco de perdermos informação importante que for a dar durante a entrevista. Caso não aceite que seja gravada, você deverá tomar notas enquanto decorre a conversa.
4. Diga também que, irá tomar notas ao longo da discussão para garantir a segurança da informação. Toda a informação gravada será confidencial e você não será identificado pelo seu nome.

Entrevistador(a) |__|__| Data: |__|__|-|__|__|-|__|__| Nr. Estudo: |__|__|-CaDMIA- FAM 0a24h -|__|__|__|-|__|__|__|

INFORMAÇÃO DEMOGRÁFICA

**Ref. Ficheiro Áudio / Número de estudo**	**|__|__|-CaDMIA-FAM 0a24h -|__|__|__|-|__|__|__|**
**Dados do participante**	**Sexo** |__| **Idade** |__|__| **Nível escolaridade:** ______________________________ **Ocupação:** ____________________________________<br>**Religião:** ____<br>**Categoria de respondente, em relação ao falecido:** ____<br>(Ex: Mãe/Pai, Esposa/Esposo, Filho, Tio, Outro)
**Dados do falecido**	**Sexo** |__| **Idade** |__|__| **M. Grávida** ☐ **Criança< 3meses** ☐ **Nado Morto** ☐<br>**Religião:** ____<br>**Data da morte:** |__|__|-|__|__|-|__|__| **Hora da morte:** |__|__|:|__|__|
**Data e local da entrevista**	|__|__|-|__|__|-|__|__| ____
**Línguas faladas**	____
**Resultado da entrevista**	☐ **Completa** ☐ **Interrompida** ☐ **Impossível de completar**<br>**Razões:** ____<br><br>**Por completar a:**<br>|__|__|-|__|__|-|__|__|

Entrevistador(a) |__|__| Data: |__|__|-|__|__|-|__|__| Nr. Estudo: |__|__|-CaDMIA- FAM 0a24h -|__|__|__|-|__|__|__|

1. CAUSA DE MORTE

**O que aconteceu? [O que causou a morte do seu familiar?]**<br><br>**Acha que é importante saber a causa de morte do seu familiar? Porquê?**<br><br>*→Pergunte quais seriam as vantagens de conhecer as causas da morte. Pergunte também as desvantagens.*<br><br>*→ Explore emoções: por exemplo, si saber a causa de morte faria/faz que a pessoa esteja mais tranquila, ou mais preocupada, etc.*	**RESUMO**

Entrevistador(a) |__|__| Data: |__|__|-|__|__|-|__|__| Nr. Estudo: |__|__|-CaDMIA- FAM 0a24h -|__|__|__|-|__|__|__|

2. MIAS

	RESUMO
**Se aqui na Manhiça (*área do estudo*) houvera uma maneira de determinar a causa de morte tirando amostra como lhe expliquei no consentimento informado (biopsia/MIA), aceitaria ou rejeitaria fazer ao seu familiar? Porquê?** <br>**Imagine que as MIAs estão a ser feitas aqui na Manhiça. Se fora um médico ou um profissional da saúde a pedir, aceitaria? Porquê?** <br>**O é que a saúde poderia fazer para facilitar para que o procedimento não prejudicasse o ritmo normal das cerimónias e para que a família não se sentisse lesada?**	

Entrevistador(a) |__|__| Data: |__|__|-|__|__|-|__|__| Nr. Estudo: |__|__|-CaDMIA- FAM 0a24h -|__|__|__|-|__|__|__|

3. O QUÉ ACONTECE QUANDO MORRE ALGUÉM?

**O quê estão a tratar neste momento?**	**RESUMO**

Entrevistador(a) |__|__| Data: |__|__|-|__|__|-|__|__| Nr. Estudo: |__|__|-CaDMIA- FAM 0a24h -|__|__|__|-|__|__|__|

OBSERVAÇÕES
