# Supplementary material for: Consent to minimally invasive tissue sampling procedures in children in Mozambique: A mixed-methods study
Source: PLoS One. 2021 Nov 8;16(11):e0259621. doi: 10.1371/journal.pone.0259621 (PMC8575303; doi:10.1371/journal.pone.0259621)
Supplement: S2 Appendix — (PDF) [file pone.0259621.s002.pdf]

cism centro de investigação em saúde de manhiça	*CENTRO DE INVESTIGAÇÀO EM SAÚDE DE MANHIÇA (CISM)*<br>**Assessment of community perceptions and the feasibility of conducting child mortality and pregnancy surveillance**<br>**OBSERVATION´S GUIDE**<br>**PHASE: Starting MITS implementation**	CHAMPS Child Health and Mortality Prevention Surveillance

A. Information about the child's death

1. How does hospital staff inform the family about the death?

a. Who is the one informing?
b. Which words are used?
c. Who is/are the one/s receiving the news?
- Family members, present and absent.
- If any close member of the family is absent, explain who, the reasons for the absence (if it's possible) and who represents him/her during her/his absence.

2. How do family members react?
- What words, expressions, gestures do they use?

3. How does hospital staff address family reactions and concerns?
- Is any non-verbal language expressing empathy, compassion, support...?
- Information, advices
- Referral/s to other services?

B. CHAMPS team preparation (from the moment of the death). [*In the pilot phase, focus on the following constructs: "feasibility", "implementation" and "ethics". Check if there is any variable that has not been listed before.*]

1. How is the death reported to the CHAMPS team?

a. Who notifies the death. To whom.
b. How? (Eg. Verbal, telephone call, telephone message, by sending other person…?)
c. What kind of documentation is used? (Eg. Forms, notes, etc.)
d. Time required for each step described above.

2. Who is involved after the death is reported?
- From the CHAMPS study. Roles and responsibilities.
- From other units/departments (From the hospital and from the CISM. Eg: Eg. Clinic, demography….). Roles and responsibilities.

3. Describe the activities taking place after the death is reported:

a. What exactly does each member of the team identified above (from the CHAMPS study and from other units/departments)? Where (Eg. At the hospital, at the CISM)?
b. Describe the interaction between CHAMPS team members (including conversations, comments, directions, decisions)

c. Describe the moments of conflict and discrepancy

d. Did any step occur outside the expected circuit? What were the reasons for this change from the *original* procedure?

C. *Time between the death is reported until "MITS team" entrance*

1. **How is the transition between the hospital staff and the CHAMPS team made, during interaction with family members?**
   a. Who makes the transition?
   b. Where does this interaction with family members take place?
   c. Are the participants in this interaction (both clinic and family) the same as in the previous steps? If not, who was absent or who was incorporated into the discussion?
   d. What is said to family members?

2. **How do family members react?**
   a. Did you notice any difference in how family members react to the CHAMPS team's input compared to how they reacted during the time the death was reported?
   b. Which questions were made by family members, if any? How did the CHAMPS 'team address those questions?

3. **Duration of this interaction (time)**

D. Sensitization, comfort, support and consent

1. **What are the main messages CHAMPS team transmits to the family when sensitizing them about the study?**

2. **Steps for obtaining informed consent**

   a. How is the study explained?
   b. Is there the presence of a witness?
   c. How are the forms used? (Eg: read, explained, handed to relatives…)
   d. How does family react?
      - Did the family ask questions? Which ones? How are those questions addressed?
      - Which family members are the most active in the consent process?
      - Role of other family members present
      - Result (acceptance or refusal)

- If acceptance, who signs the consent? (relation with the child)
- If refusal, which ones are the family´s main concerns? How does the CHAMPS team react?

3. How is comfort and support offered to the family?

a. Type of support [Eg. Emotional, religious, material, logistic, informative/scientific…]

4. Does the CHAMPS team provide some additional resources?

a. Informative/educative materials?
b. Referrals to other services?

E. MITS

1. During the entire process (from the child´s death to request for consent), where is the body of the child?

2. After consent is obtained, what specific activities happen next?

a. Who is responsible for each (both from the family and the staff´s side)?
b. Have the family members the chance to see the body before MITS?
c. Where is the body transported for conducting MITS?
d. Who performs the MITS procedure?
e. Does any family member (or other person but the CHAMPS team) participates or observe the MITS procedure?
f. Where are the family members during the procedure?
g. How long did the procedure last?

3. Timing:

a. How long does it take from obtaining consent until:
    - The MITS is carried out
    - Any other clinical procedures are carried out
    - The body is returned to the family
b. Does the family demonstrate any problems with the timing?

[CONSTRUCTS: IMPLEMENTATION; PRACTICALITY; ETHICAL CONSIDERATIONS; GRIEVING AND MOURNING]

Modes of communication to family

a. Which RPRRU staff talks to the family? What information do they communicate?
b. Which family member is involved in the phone conversation?
c. What are the modes of communication? (e.g., phone, in-person)?
d. If possible to observe, what are the family's interactions?
e. Throughout the course of CHAMPS activities, are other forms of communication employed (e.g. written notifications), and for what purposes?

[CONSTRUCTS: IMPLEMENTATION; GRIEVING AND MOURNING; PRACTICALITY]

Body release

a. What are the processes for returning the body to the family?
b. Who on the RMPRU staff is responsible for this?
c. Who is the family counterpart?
d. How does the family interact?
    - Among themselves?
    - With the staff?
e. What are the immediate concerns from the family perspective and how are they voiced to each other or the staff?
    - Transport
    - Money
    - Delays in ceremonies
    - Incentives

[CONSTRUCTS: GRIEVING AND MOURNING; ETHICAL CONSIDERATIONS; IMPLEMENTATION; ACCEPTABILITY]

Family follow-up

a. Where do the follow-up meetings take place and who is involved?
b. What is communicated to the family during follow up meetings?
c. What RMPRU staff are part of these meetings?
d. What are their specific roles?
e. How does the family interact?
