# Supplementary material for: Consent to minimally invasive tissue sampling procedures in children in Mozambique: A mixed-methods study
Source: PLoS One. 2021 Nov 8;16(11):e0259621. doi: 10.1371/journal.pone.0259621 (PMC8575303; doi:10.1371/journal.pone.0259621)
Supplement: S3 Appendix — (PDF) [file pone.0259621.s003.pdf]

CENTRO DE INVESTIGAÇÀO EM SAÚDE DE MANHIÇA (CISM)

Avaliação das Percepções da Comunidade e Viabilidade de um Programa de Vigilância de Mortalidade Neonatal e Infantil no Distrito de Manhiça (Estudo CHAMPS-SBS)

Stage 2

GUIÃO DE ENTREVISTA SEMI-ESTRUTURADA

Familiares de crianças falecidas que aceitaram a realização da MITS

Instruções

1. Explique ao entrevistado que:
   - Esta entrevista tem como objectivo conversar com os participantes sobre o programa (estudo) CHAMPS e aspetos relacionados com a recolha de amostras de tecidos e fluidos no corpo de crianças recém-falecidas de forma minimamente invasiva (MITS).
   - Foi convidado (a) para esta entrevista porque devido a sua experiência recente com CHAMPS e MITS, depois do falecimento da sua criança, a sua contribuição é essencial para entender os tópicos acima, desde a perspetiva da família.
   - Vamos iniciar a nossa conversa com questões relacionadas com o consentimento informado. Depois, vamos continuar com aspectos relacionados com a recolha de amostras de tecido e fluídos no corpo de crianças recém-falecidos. Finalmente vamos falar sobre os resultados derivados da análise destas amostras de tecidos e fluídos e sobre sua entrega aos familiares das crianças falecidas. Espera-se que a entrevista dure 40 minutos- 1 hora.

2. Diga que, se permitir, a entrevista será gravada, somente para não corrermos o risco de perdermos informação importante que for a dar durante a entrevista. Caso não aceite que seja gravada, o entrevistador tomará notas enquanto decorre a conversa.

3. Diga também que, embora a entrevista seja gravada, também irá tomar notas ao longo da conversa para garantir a segurança da informação. Toda a informação gravada será confidencial e você não será identificado (a) pelo seu nome.

INFORMAÇÃO DEMOGRÁFICA

**Ref. Ficheiro Áudio / Número do Estudo**	**CHAMPS_MZ_SSI_FM_|__|__|__|__| - |__|__|__|__|__|__|__|** (ID arquivo) (ID do particip- Nº e 3 letras)
**Dados do participante**	Sexo |__| Idade |__|__| Natural de (província e localidade): ____________________<br>____<br>Mora em (P. Admin. e bairro): ______________________________<br>Nível escolaridade (último nível concluído): ______________________<br>Ocupação: ______________________ Estado civil: ________________<br>Religião e igreja: ______________________________________<br>Categoria do respondente em relação ao falecido (Ex. mãe/pai, tio/a, avô/avó, irmão/irmã...):<br>______________________________
**Dados do falecido**	Sexo|__| Idade |__|__| __________ (dias/meses/anos)<br>Data da morte |__|__|/|__|__|/|__|__|__|__| Nado morto ☐ Neonato ☐ Aborto ☐
**Data e local da entrevista**	|__|__|/|__|__|/|__|__|__|__| ______________________________
**Línguas faladas**	____
**Resultado da entrevista**	Gravada ☐ Não gravada ☐ Motivo:____________________<br>______________________________<br>Completa ☐ Interrompida ☐ Motivo: __________________<br>______________________________<br>Impossível de completar ☐<br>Por completar em (data):<br>____________________
**Entrevistador: |__|__|__|**	

1. MITS

	RESUMO
1. Pode me explicar como morreu a criança?<br>*[Brevemente. Trata-se de saber como morreu a criança, onde buscaram tratamento, e o que aconteceu na unidade sanitária]*<br><br>2. Pode me explicar de que forma é que pediram para realizar MITS na criança falecida?<br><br>→ *Se a resposta for não sabe ou não foi feito um pedido, pergunte*<br>• *Como gostaria que tivesse sido feito o pedido*<br><br>*3.* O que acha sobre o pedido de consentimento que foi feito para realização da MITS na criança falecida? Porquê?<br><br>Explore o que o entrevistado acha sobre:<br><br>➔ A forma como foi realizado o pedido de consentimento<br>➔ O momento e lugar em que se pede/pediu o consentimento<br>➔ O tempo que se leva a pedir o consentimento<br>➔ A pessoa que pede ou pediu o consentimento<br>➔ Se o pedido foi feito a pessoa certa<br>➔ A informação transmitida<br>➔ A melhor forma (como devia ser realizado?)	

4. Como é que se chegou à decisão de realizar MITS?<br><br>Explore:<br><br>➔ Motivos que lhes levaram a aceitar MITS?<br>➔ Pessoas envolvidas?<br>➔ Pessoas que tiveram a última palavra?<br>➔ Dificuldades para tomar a decisão?<br><br>5. O que acha sobre a MITS que foi realizado no corpo da criança falecida?<br><br>Explore o que o entrevistado acha sobre:<br>➔ O tempo de duração da MITS<br>➔ Lugar onde se fez MITS<br>➔ Os profissionais de saúde envolvidos<br>➔ O estado em que foi devolvido o corpo<br>➔ O que se falou no consentimento informado, foi o que aconteceu<br><br>6. Acha que alguém da família deveria ter acompanhado a realização da MITS? ***Se sim***, quem? Porquê? E **se não**, porque não?<br><br>7. A quem da família informaram que se fez MITS na criança? Quando informaram? E como reagiram? (emoções, sentimentos, comentários da família)	

8. A realização de MITS afetou programa para realização do funeral da criança falecida? Como?<br><br>• Tiveram algum custo adicional por ter realizado MITS na criança? Ou um serviço que não tiveram que pagar pelo facto de fazer MITS na criança?<br><br>9. Acha que a MITS no corpo da criança falecida podia ter sido realizada em sua casa? Porquê?<br>• O que acha de se realizar MITS na sua comunidade?<br><br>➔ Em que lugares na sua comunidade pode-se realizar MITS?<br>➔ Que barreiras e/ou dificuldades haveria para se realizar MITS na sua comunidade?<br>➔ O que facilitaria para que se realizasse MITS na sua comunidade?<br><br>10. Como é que o Programa CHAMPS poderia melhorar para que tudo o que falamos até agora seja feito da melhor forma?	

2. ENTREGA DE RESULTADOS DAS MITS

1. Já recebeu resultados de MITS?<br>➔ ***Se sim***, faça perguntas do quadro/secção número 3<br>➔ ***Se não,*** siga com as perguntas abaixo.	**RESUMO**

2. O que acha sobre a entrega de resultados derivado da MITS que foi feita a criança falecida?<br><br>Explore :<br><br>➔ Gostaria de receber os resultados?<br>➔ Se a equipa contatou com ele/a, e se tem informação de quando e como vão ser entregues.<br><br>➔ O tempo de espera pelos resultados?<br>➔ Pessoas envolvidas (quem dá e quem recebe os resultados)<br><br>3. O que pode mudar ou mudou por saber da doença ou causa que matou a sua criança?<br><br>4. Você ou algum familiar buscou respostas sobre a causa de morte em algum outro lugar? Onde? [Ou consultou a alguém sobre a causa de morte da criança?]<br><br>5. Alguém do seu meio lhe sugeriu alguma causa de morte da criança? Quem?	

3. RESULTADOS DAS MITS

	RESUMO
1. O que acha sobre a entrega de resultados derivado da MITS que foi feita a criança falecida?<br><br>Explore :<br>➔ O que pensa sobre o tempo que levou à espera pelos resultados de MITS feita ao corpo da criança falecida?<br>➔ Se a equipa contatou com ele/a e deu informação de quando e como vão ser entregues.<br>➔ O que pensa sobre as pessoas envolvidas na entrega de resultados (quem dá e quem recebe os resultados)<br>➔ Linguagem utilizada?<br><br>2. O que acha dos resultados que recebeu da sua criança falecida? Porquê?<br><br>3. O que pensa em fazer com os resultados que recebeu da criança falecida? Porquê?<br><br>4. O que pode mudar ou mudou por saber da doença ou causa que matou a sua criança?	

5. Buscou respostas sobre a causa de morte em algum outro lugar? Onde? *[Ou consultou a alguém sobre a causa de morte da criança?]*<br><br>6. Alguém do seu meio lhe sugeriu alguma causa de morte da criança? Quem?	

Há algum tema que surgiu que deva se pôr em conhecimento de outras equipas CHAMPS (CE, Clínica, Demografia...)?

____
____
____
____
____
____

OBSERVAÇÕES:

____
____
____
____
____
____
____
____

____
____
____
____
____
____
____
____
____
____
____
____
____
____
____
____
____
____
____
____
____
____
