# Supplementary material for: Consent to minimally invasive tissue sampling procedures in children in Mozambique: A mixed-methods study
Source: PLoS One. 2021 Nov 8;16(11):e0259621. doi: 10.1371/journal.pone.0259621 (PMC8575303; doi:10.1371/journal.pone.0259621)
Supplement: S4 Appendix — (PDF) [file pone.0259621.s004.pdf]

cism centro de investigação em saúde de manhiça	*CENTRO DE INVESTIGAÇÀO EM SAÚDE DE MANHIÇA (CISM)*<br>**Avaliação das Percepções da Comunidade e Viabilidade de um Programa de Vigilância de Mortalidade Neonatal e Infantil no Distrito de Manhiça (Estudo CHAMPS-SBS)**<br>**Stage 2**<br>**GUIÃO DE ENTREVISTA SEMI-ESTRUTURADA**<br>**Familiares de crianças falecidas que recusaram a realização da MITS**	CHAMPS Child Health and Mortality Prevention Surveillance

Instruções

1. Explique ao entrevistado que:
    - Esta entrevista tem como objectivo conversar com os participantes sobre CHAMPS e aspetos relacionados com a recolha de amostras de tecidos e fluidos no corpo de crianças recém-falecidas de forma minimamente invasiva (MITS).
    - Foi convidado (a) para esta entrevista porque devido a sua experiência recente com CHAMPS e MITS, a sua contribuição é essencial para entender os tópicos acima, desde a perspetiva da família.
    - Vamos iniciar a nossa conversa com questões relacionadas com o consentimento informado. Depois, vamos continuar com aspectos relacionados com a recolha de amostras de tecido e fluídos no corpo de crianças recém-falecidos. Finalmente vamos falar sobre a entrega dos resultados aos familiares das crianças falecidas e sobre acausa de morte da criança falecida. Espera-se que a entrevista dure 1h a 1h e 30 minutos.

2. Diga que, se permitir, a entrevista será gravada, somente para não corrermos o risco de perdermos informação importante que for a dar durante a entrevista. Caso não aceite que seja gravada, o entrevistador tomará notas enquanto decorre a conversa.

3. Diga também que, irá tomar notas ao longo da conversa para garantir a segurança da informação. Toda a informação gravada será confidencial e você não será identificado (a) pelo seu nome.

INFORMAÇÃO DEMOGRÁFICA

**Ref. Ficheiro Áudio / Número do Estudo**	**CHAMPS_MZ_SSI_FM_**☐☐☐☐_☐☐☐☐☐☐☐<br>(ID arquivo) (ID do particip- Nº e 3 letras)
**Dados do participante**	Sexo ☐ Idade ☐☐ Natural de (província e localidade): ____________________________<br>____<br>Mora em (P. Admin. e bairro): ____<br>Nível escolaridade (último nível concluído): __________________________________<br>Ocupação ____________________________________Estado civil_______________<br>Religião e igreja: ____<br>**Categoria do respondente em relação ao falecido** (Ex. mãe/pai, tio/a, avô/avó, irmão/irmã...)<br>____
**Dados do falecido**	**Sexo**☐ Idade ☐☐__________ (dias/meses/anos)<br>**Data da morte** ☐☐/☐☐/☐☐☐☐<br>Nado morto ☐ Neonato ☐ Aborto ☐
**Data e local da entrevista**	|__|__|-|__|__|-|__|__|__|__| ____
**Línguas faladas**	____
**Resultado da entrevista**	**Gravada** ☐ **Não gravada** ☐ **Motivo:**_____________________<br>____<br>**Completa** ☐ **Interrompida** ☐ **Motivo:** ____________________<br>____<br>**Impossível de completar** ☐<br>**Por completar em (data):**<br>__________________________
**Entrevistador: |__|__|__|**	

1. MITS

	RESUMO
1 . O que acha sobre o pedido de consentimento que foi feito para realização da MITS na criança falecida? Porquê?<br><br>Explore o que o entrevistado acha sobre:<br><br>➔ A forma como foi realizado o pedido de consentimento?<br>➔ A melhor forma de pedir o consentimento?<br>➔ O momento em que se pede/pediu o consentimento?<br>➔ A pessoa que pede ou pediu o consentimento?<br>➔ Se o pedido foi feito a pessoa certa?<br><br>2. Como é que se chegou à decisão de recusarem a realização da MITS?<br><br>Explore:<br><br>➔ Pessoas envolvidas?<br>➔ Pessoas que tiveram a última palavra?<br>➔ Dificuldades para tomar a decisão?<br>➔ Motivos que lhes levaram a recusar MITS?	

3. De que forma a acha que a realização de MITS teria interferido no programa para realização do funeral da criança falecida?<br><br>Explore:<br>➔ Que programas ou cerimónias teriam sido mais afectados?<br><br>4. Aconselharia uma família que perdeu uma criança a realizar a MITS? Porquê?<br><br>Explore:<br>➔ O que poderia dizer a família como um conselho para aceitar realizar a MITS?<br><br>5. O que acha sobre realizar MITS em casa ou num outro sítio na sua comunidade?<br><br>Explore o que o entrevistado acha sobre:<br>➔ Pessoas da comunidade assistirem a realização da MITS? Que pessoas da comunidade poderiam ser envolvidas?<br>➔ Barreiras e dificuldades?	

2. ENTREGA DE RESULTADOS DAS MITS

1. Se tivesse aceite realizar MITS na criança falecida, como CHAMPS deveria fazer a entrega dos resultados?<br><br>Explore o que o entrevistado acha sobre:<br><br>➔ O tempo que a familia poderia esperar para receber os resultados?<br>➔ A forma como a familia devia ser contactado e informado?<br>➔ Pessoas que seriam envolvidas na divulgação dos resultados?<br>➔ Dificuldades e barreiras para fazer a entrega?	**RESUMO**

3 . Causa da morte

1. Preocupou-se em procurar saber da causa da morte da criança? Porquê?<br><br>Explore:<br>➔ Aonde é que procurou saber da causa da morte?<br>➔ Depois de quanto tempo ter ocorrido a morte da criança?<br>➔ Quem são as pessoas que tomaram essa decisão?<br>➔ O que acha da causa de morte que descobriu?<br>➔ Dificuldades encontradas para descobrir a causa de morte?	**RESUMO**

Há algum tema que surgiu que deva se pôr em conhecimento de outras equipas CHAMPS (CE, Clínica, Demografia...)?

____
____
____
____
____
____

OBSERVAÇÕES:

____
____
____
____
____
____
____
____
____
____
____
____
____
____
____
____
____
____
____
